# New use of an old drug: mechanism of oseltamivir phosphate inhibiting liver cancer through regulation of lipophagy via NEU1

**DOI:** 10.3389/fphar.2025.1556661

**Published:** 2025-03-24

**Authors:** Yuyu Chen, Peiyu Han, Haixia Zhu, Wenchao Zhang, Xiaoyu Ma, Yiting He, Hetian Chen, Weiwei He, Yu Wu, Yuqiu Ge

**Affiliations:** ^1^ MOE Medical Basic Research Innovation Center for Gut Microbiota and Chronic Diseases, Wuxi School of Medicine, Jiangnan University, Wuxi, China; ^2^ Department of Nuclear Medicine, Affiliated Hospital of Jiangnan University, Wuxi, China; ^3^ Clinical Laboratory, Tumor Hospital Affiliated to Nantong University, Nantong, China; ^4^ Nanjing Hospital of Chinese Medicine, Affiliated to Nanjing University of Chinese Medicine, Nanjing, China

**Keywords:** NEU1, liver cancer, lipophagy, PLIN2, oseltamivir phosphate

## Abstract

**Background:**

Neuraminidase-1 (NEU1) is an enzyme that breaks down sialic acids on glycoproteins and glycolipids. Aberrant expression of NEU1 has been linked to the progression of numerous malignancies, including liver cancer. Oseltamivir phosphate (OP) is a drug used to treat and prevent influenza, which specifically inhibits NEU1. However, the molecular mechanisms of NEU1 in liver cancer and the potential therapeutic effects of OP remain largely unclear.

**Methods:**

NEU1 expression in liver cancer was evaluated using public databases and validated in our samples. CRISPR/Cas9, CCK-8 assay, transwell assays, oil red O staining, RNA-sequencing, immunofluorescence and co-immunoprecipitation (Co-IP) and *in vivo* experiments were used to investigate the biological function of NEU1 and the therapeutic effect of OP in liver cancer.

**Results:**

We demonstrated that NEU1 expression was significantly elevated in liver cancer cells and tumor tissues. Patients with liver cancer exhibiting high levels of NEU1 expression tended to have a less favorable prognosis. NEU1 knockdown inhibited liver cancer cells proliferation, invasion and migration. Subsequent experiments demonstrated that NEU1 knockdown reduced lipid accumulation through promoting perilipin 2 (PLIN2)-mediated lipophagy. Notably, OP (NEU1 inhibitor), promoted lipophagy, thereby inhibiting liver cancer proliferation and tumorigenesis. Moreover, liver cancer cells were more sensitive to OP compared to other chemotherapeutics, like 5-fluorouracil and gemcitabine, with a reduced drug resistance.

**Conclusion:**

OP inhibits liver cancer progression by targeting NEU1 and inducing lipophagy through the suppression of PLIN2. Our findings provide new directions on the role of NEU1 in liver cancer and offer latent strategies to address the chemotherapy-induced drug resistance.

## 1 Introduction

Liver cancer is an aggressive malignant tumor with a challenging early diagnosis, characterized by rapid progression, high rates of recurrence, and metastasis. This results in a poor prognosis with a 5-year survival rate ranging from 15% to 38% (Ye et al., 2016). The 2020 global cancer statistics highlight that liver cancer has notably exacerbated the global burden of cancer-related mortality (Sung et al., 2021). The majority of patients with liver cancer are diagnosed in the middle to late stages, often with metastasis, rendering them unsuitable for surgical treatment (Forner et al., 2018; Wang and Deng, 2023). Consequently, chemotherapy has emerged as the primary treatment option. However, resistance to chemotherapeutic agents is a major challenge in liver cancer treatment, leading to frequent clinical treatment failures (Weiwei et al., 2020; Junru et al., 2022). Therefore, there is urgently needed to explore the underlying mechanisms driving the progression and metastasis of liver cancer, reduce chemotherapy resistance, and enhance the effectiveness of chemotherapy.

Neuraminidase-1 (NEU1) is an essential enzyme involved in the breakdown of sialic acid on cell surface and plays multiple roles beyond lysosomes catabolism, including immune response signaling and elastic fiber assembly (Pshezhetsky and Hinek, 2011). Studies have linked NEU1 in several diseases including diabetes (Blaise et al., 2013), ovarian cancer (Ren et al., 2016) and bladder cancer (Zhou et al., 2020). In tumor progression, elevated NEU1 levels are often associated with increased tumor aggressiveness, chemotherapy resistance, and immune evasion, prompting exploration of NEU1 as a potential therapeutic target (Gorelik et al., 2023). Liver cancer, being a metabolism-related disease, has shown a close association with NEU1 through recent bioinformatics analysis of The Gene Expression Omnibus (GEO) and The Cancer Genome Atlas (TCGA) datasets (Wu et al., 2021). However, laboratory evidence remains limited, and further investigation is necessary to elucidate the molecular mechanism of NEU1 regulating liver cancer.

It is noteworthy that oseltamivir phosphate (OP), a sialidase inhibitor, effectively cut off and inhibit infected cells, and is currently widely used in the influenza treatment. The therapeutic applications of OP have been expanded in recent studies. Qin Y et al. revealed the OP’s beneficial effect on the cardiac health by targeting NEU1, which inhibits mitochondrial fission and mitophagy mediated by dynamin-associated protein 1, thereby improving cardiac dysfunction in doxorubicin-induced cardiomyopathy (Qin et al., 2021). The potential of OP as a NEU1 inhibitor in cancer therapy is being increasing explored. Studies have shown that OP inhibits breast cancer cells proliferation (Thulasiraman et al., 2019; Haxho et al., 2014; Sambi et al., 2020), and displays potential as a therapeutic agent for drug-resistant pancreatic cancer (O’Shea et al., 2014; Itakura and Mizushima, 2011). In addition, OP enhanced the sensitivity of esophageal squamous cell cancer cells to 5-fluorouraeil (5-FU), showing great potential in cancer treatment (Yang et al., 2024). Recent bioinformatics analysis have confirmed NEU1’s role in carcinogenesis of liver cancer (Wu et al., 2021). More importantly, Huang et al. found that OP can induce apoptosis and autophagy of HepG2 cells, demonstrating exert anti-liver cancer activity (Huang et al., 2021). Lipophagy is a major form of autophagy that relies on autophagy-lysosomal pathway to engulf and degrade fats, polysaccharides and proteins. Evolved as self-protection mechanism, lipophagy helps maintain lipid homeostasis and manage metabolic waste by processing intracellular lipids and other substances (Singh et al., 2009; Zechner et al., 2017; Zhang et al., 2022). Abnormalities in lipid metabolism are often associated with liver cancer development. Emerging researches have highlighted the significant role of lipophagy in liver diseases, including non-alcoholic fatty liver disease and alcoholic fatty liver disease (Grefhorst et al., 2020; Lin et al., 2013).

Despite these advances, the specific mechanisms by which NEU1 participates in the occurrence and progression of liver cancer, particularly the role of lipophagy, as well as the potential therapeutic effects of its inhibitor, OP, remain underexplored. In this study, we utilized public databases such as Gene Expression Profiling Interactive Analysis (GEPIA) and Kaplan-Meier Plotter, along with data from our own cohort, to investigate the expression patterns and prognostic value of NEU1 in liver cancer. We then assessed the impact of NEU1 knockdown on the malignant phenotype of liver cancer cells and explored the underlying mechanisms through RNA sequencing, Oil Red O staining, and co-immunoprecipitation. Additionally, both *in vitro* and *in vivo* studies were conducted to evaluate the anti-liver cancer effects of OP by targeting NEU1. Our findings offer novel insights into how NEU1 regulates lipophagy by binding to perilipin 2 (PLIN2), influencing liver cancer progression. Furthermore, we demonstrate that inhibiting NEU1 with OP could serve as a potential therapeutic strategy for liver cancer treatment in the future.

## 2 Material and methods

### 2.1 Analysis of NEU1 expression in public databases

cBioPortal can be used to explore, multidimensional cancer genome data visualization and analysis (de Bruijn et al., 2023). In our study, we analyzed NEU1 expression in a variety of tumors. The expression levels of NEU1 in liver cancer was evaluated, and its correlation with clinicopathological parameters was assessed using GEPIA platform, which is based on TCGA the liver hepatocellular carcinoma dataset (http://gepia2.cancer-pku.cn). Besides, the impact of NEU1 expression on overall survival and disease-free survival were analyzed in the Kaplan-Meier plotter. Survival curves were plotted, and the hazard ratio along with the 95% confidence interval (95% CI) was calculated by a cox regression model (https://kmplot.com). In addition, the GEO database (https://www.ncbi.nlm.nih.gov/geo/) was used to validate the expression characteristics of NEU1 in liver cancer, including datasets GSE55092, GSE121248, GSE45436, GSE62232, GSE101685, and GSE76427.

### 2.2 Cell culture and transfection

Human normal liver cell lines (LO2, QSG-7701) and human liver cancer cell lines (HepG2, Huh7, MHCC97H) were procured from the Shanghai Institute of Cell Biology, Chinese Academy of Sciences. HepG2, LO2 and QSG-7701 cells were cultured in RPMI-1640 (Hyclone, United States) supplemented with 10% fetal bovine serum (FBS, Invitrogen, United States) and 1% penicillin and streptomycin. Huh7 and MHCC97H cells were maintained in DMEM (Gibco, United States) under the same condition. For transfection experiments, plasmids were introduced into the cells using Lipofectamine 2000 reagent (Beyotime Biotechnology, China, TL201-01) following the manufacturer’s protocol. The resulting cell lines, expressing Cas9-NC, Cas9-NEU1, pCDH-NC, and pCDH-PLIN2 were obtained. Plasmids for NEU1 knockdown or overexpressing PLIN2 were constructed by Plasmid Inc (Vector Builder). The plasmid sgRNA sequences are as follows: Cas9-*NEU1*-1: CTT​GGC​CCC​CTC​ATC​GGA​TG; Cas9-*NEU1*-2: AAG​GGC​CGC​CTC​ATC​GTG​TG.

### 2.3 Western blot

Cells were lysed with RIPA buffer (Beyotime Biotechnology, Shanghai, China), protease inhibitors and phosphatase inhibitors (Beyotime Biotechnology, Shanghai, China) mixture, and the supernatant was extracted. The proteins were extracted, separated by SDS-PAGE, and transferred to PVDF membranes. The membranes were then blocked with 5% skim milk and incubated overnight with primary antibodies. Visualization was achieved using the ECL kit (Vazyme, Nanjing, China). The antibodies used for protein analysis were as follows: anti-NEU1 (1:1000, 67032-1-Ig, Proteintech, Chicago, United States), anti-PLIN2 (1:500, ET1704-17, HUABIO, Hangzhou, China), anti-Ki67 (1:1000, Proteintech, 28074-1-AP, Chicago, United States), anti-Vimentin (5741), anti-E-cadherin (14472), anti-PCNA (13110), anti-LC3B-II (4108), anti-GAPDH (5174) or β-actin (4970) were purchased from Cell Signaling Technology (United States), the dilution concentration was 1:1000.

### 2.4 Quantitative real-time PCR

Total RNA was extracted from cells using TRIzol Regent (CWBIO, Jiangsu, China) following a previously established protocol. Subsequently, the extracted RNA was reverse-transcribed into cDNA using HiScript III All-in-one RT SuperMix Perfect for qPCR (Vazyme, Nanjing, China). qRT-PCR was performed on a LightCycler 480 II (Roche, United States) using Chamq SYBR qpcr Master mix (Vazyme, Nanjing, China). The relative RNA levels in each experimental group were determined using the 2^−ΔΔCt^ method, with normalization to GAPDH, which served as internal controls. The primers sequences used for qRT-PCR are provided below: *NEU1*-Forward: 5′-GGA​GGC​TGT​AGG​GTT​TGG​G-3′; *NEU1*-Reverse: 5′-CAC​CAG​ACC​GAA​GTC​GTT​CT-3′; *GAPDH*-Forward: 5′-AGG​TCG​GTG​TGA​ACG​GAT​TTG-3′; *GAPDH*-Reverse: 5′-CAC​CAG​ACC​GAA​GTC​GTT​CT-3′.

### 2.5 Immunohistochemistry staining

Immunohistochemical staining was conducted on liver cancer samples, including both cancerous and adjacent tissues, as well as tumor samples from xenograft mice, following established protocols. Informed consent was obtained from all patients and volunteers involved, and this study was approval by Jiangnan University (JNU202409RB0012). Tumor specimens were immersed in 4% paraformaldehyde. Paraffin embedded tissues were sectioned, and the thickness of the sections was 4 µm. Following dewaxing, rehydration, and antigen retrieval steps, the slides were incubated with specific primary antibodies against NEU1, PLIN2 and LC3B-II. Then, the sections were incubated with the corresponding secondary antibody, followed by dyeing with DAB (CWBIO, Taizhou, China) and counterstaining with hematoxylin. Representative images were captured using Pannoramic MIDI (3DHISTECH, Budapest, Hungary).

### 2.6 Immunofluorescence analysis

HepG2 and MHCC97H cells were inoculated in 24-well plates fitted with glass slides and allowed to adhere and reach approximately 60% confluence. Following either 24 h of plasmid transfection or treatment with OP, the cells and lipoprobes were incubated with at 37°C away from light, washed, fixed, permeabilized, and sealed with bovine serum albumin. Next, the cells were incubated with antibodies targeting NEU1, PLIN2, or LC3B-II (diluted at 1:100). The sample at a temperature of 4°C was incubated for overnight, the cells were incubated with secondary antibodies conjugated with 649 (red), Alexa Fluor 488 (green) to detect the localization and expression of target proteins. Finally, the slides were sealed with DAPI, and immunofluorescence images were acquired using confocal microscopy. Uniform cell distribution fields were selected using Zen software (Zeiss, 2024. ZEN Software: Features and Applications. Carl Zeiss AG. https://www.zeiss.com/microscopy/zh/products/software/zeiss-zen.html), and the mean fluorescence intensity of cells in the same field was quantified using ImageJ (Schneider et al., 2012).

### 2.7 Co-immunoprecipitation

HepG2 cells were seeded on 10 cm Petri dishes and transfected with plasmids. The specific antibodies and anti-IgG were incubated with protein A/G beads (Abbkine, Wuhan, China) for 1 h at room temperature. The supernatant of lysed cells was incubated overnight at 4°C with protein A/G beads (Abbkine, Wuhan, China). The immunoprecipitates were then separated by SDS-PAGE. Finally, we applied Western blot to verify the immunoprecipitation of NEU1 and its interaction with PLIN2.

### 2.8 Cell proliferation and colony formation assays

A cell suspension with a concentration of 8,000 cells per 100 μL was prepared and transferred to a 96-well plate. The absorbance of cells at 450 nm was measured using CCK-8 assay (Beyotime Biotechnology, Shanghai, China) and the proliferation rate was calculated. For the colony formation assay, 1,000 to 1,500 cells were plated in 6-well plates and incubated for around 14 days. Afterward, the colonies were fixed with 4% formaldehyde and stained with 0.1% crystal violet for 10 min. The number of colonies was then counted to evaluate the colony-forming ability of the cells.

### 2.9 Cell invasion and migration

Invasion assays were performed based on previous studies. In brief, 5 × 10^4^ cells in 200 µL of serum-free medium were inoculated in a transwell insert (Corning, Shanghai, China) coated with matrigel (Corning, Shanghai, China). In the lower chamber of 24-well plate, 600 µL of medium supplemented with 10% FBS was added. Following a 48-h at 37°C, the migrated cells were fixed with 4% paraformaldehyde and stained with 0.1% crystal violet solution. Subsequently, the migrated cells were imaged and counted using an Olympus microscope.

For the scratch assay, a total of 3 × 10^5^ cells were seeded into 6-well plates and incubated until reaching confluence. Cells were transfected with plasmid or treated with drugs. A scratch test was then performed by scraping the bottom of the 6-well plate with a P-100 pipette tip, and the suspended cells were gently washed with PBS. The cells were subsequently cultured in medium containing 1% FBS for 48 h at 37°C in a humidified incubator with 5% CO_2_. The scratch site was imaged using a microscope with a digital camera at 0, 24 and 48 h. Images were acquired at the designated time points, and the migration distance was calculated.

### 2.10 Oil red O staining

After treating cells in 24-well plates with climbing slides, the medium was aspirated off and washed with PBS. Cells were fixed with 4% paraformaldehyde for 20 min, then washed with PBS. Subsequently, they were moisturized and washed with 60% isopropanol for 30 s. Each well was then filled with 1–2 mL of oil red O staining solution (NanJing JianCheng Bioengineering Institute, Nanjing, China), and filtered by a 0.22 μm filter and stained for 30 min at room temperature in darkness. The oil red O staining solution was then aspirated, and the wells were rinsed with 60% isopropanol, followed by a light staining with hematoxylin for 1 min. After PBS washing, the slides were sealed. The lipid droplets were specifically stained red, while the nuclei were stained blue, observable under an inverted microscope. For lipid droplet number quantification, we utilized Image-Pro Plus 6.0 (IPP 6.0) software (Kong et al., 2019). Fields with uniform cell distribution were selected, and the software was used to determine the average number of lipid droplets per cell within the same field.

### 2.11 ZDOCK analysis

ZDOCK is a computational tool for protein-protein molecular docking, designed to simulate and predict the interaction between two protein molecules and their binding sites (https://zdock.umassmed.edu/) (Pierce et al., 2014). The 3D structures of NEU1 (PDB ID: 8DU5) and PLIN2 (PDB ID: 3EWO) proteins were obtained from the Protein Data Bank (https://www.rcsb.org/) and the interacting protein domains were predicted using the ZDOCK server and PDBePISA server (https://www.ebi.ac.uk/pdbe/pisa/). These structures were then visualized by the molecular graphics software Jmol (http://www.jmol.org/).

### 2.12 Reagent treatment

Oseltamivir phosphate (OP) 75 mg capsules were dissolved in 10 mL sterile 1× PBS and centrifuged at 1,000 rpm for 10 min. The stock-extracted solution of OP had a concentration of 18 mM. Various concentrations of OP solutions were used in cell culture for both *in vitro* and *in vivo* experiments.

Gemcitabine hydrochloride (GEM) (Sigma-Aldrich, St. Louis, MO, United States) was dissolved in phosphate-buffered saline to prepare a 50 mg/mL stock solution. This stock solution was then serially diluted to achieve GEM concentrations of 0.5, 1, 2, 4, and 8 μg/mL in culture medium containing 10% FBS and 1% penicillin-streptomycin solution. The resulting solutions were added to Petri dishes or 96-well plates with each medium change.

5-FU (Sigma-Aldrich, St. Louis, MO, United States) was dissolved in dimethyl sulfoxide to create a 98 μg/mL stock solution. This stock was serially diluted to produce various concentrations of 5-FU (1–20 μg/mL) in culture medium containing 10% FBS and 1% penicillin-streptomycin solution. The diluted solutions were added to Petri dishes or 96-well plates with each medium change.

### 2.13 Animal experiments

The animals were maintained in a pathogen-free environment, and all animal experiments were conducted in accordance with the protocols approved by the Animal Care Committee of Jiangnan University and following the guidelines of the Animal Protection and Use Committee [JN. No20230515b0560805 (222)]. MHCC97H cells (5 × 10^7^/mL) were injected into the same flank of athymic male BALB/c nude mice, which were 5 weeks old and obtained from Gempharmatech (Nanjing, China). Each group consisted of 5 mice. Tumor progression was closely monitored by measuring tumor volume, calculated using the formula (length × width^2^)/2. When the largest tumor reached a size of 1.0 cm^3^, all mice were humanely euthanized, and tumors, livers, and lungs tissues were collected for further analysis. Additionally, tumor tissues were collected for Western blot and qRT-PCR analysis. For *in vivo* imaging of tumors and lungs tissues in mice, fluorescence intensity was quantified using ImageJ software (Schneider et al., 2012).

### 2.14 Statistical analysis

Data are shown as the mean ± standard deviation (SD). The differences of continuous variables between two groups were compared by Student’s t-test, following a test for homogeneity of variances. For comparisons across multiple groups, One-way ANOVA was applied. The survival curve was generated using Kaplan-Meier analysis, and the difference was determined by the log-rank test. Statistical analyses were performed using GraphPad Prism 8.0 (GraphPad Software Inc., San Diego, CA, United States) (Berkman et al., 2019). Statistical significance was set at *P* < 0.05.

## 3 Results

### 3.1 NEU1 is upregulated in liver cancer and linked to the prognosis of liver cancer patients

**FIGURE 1:**
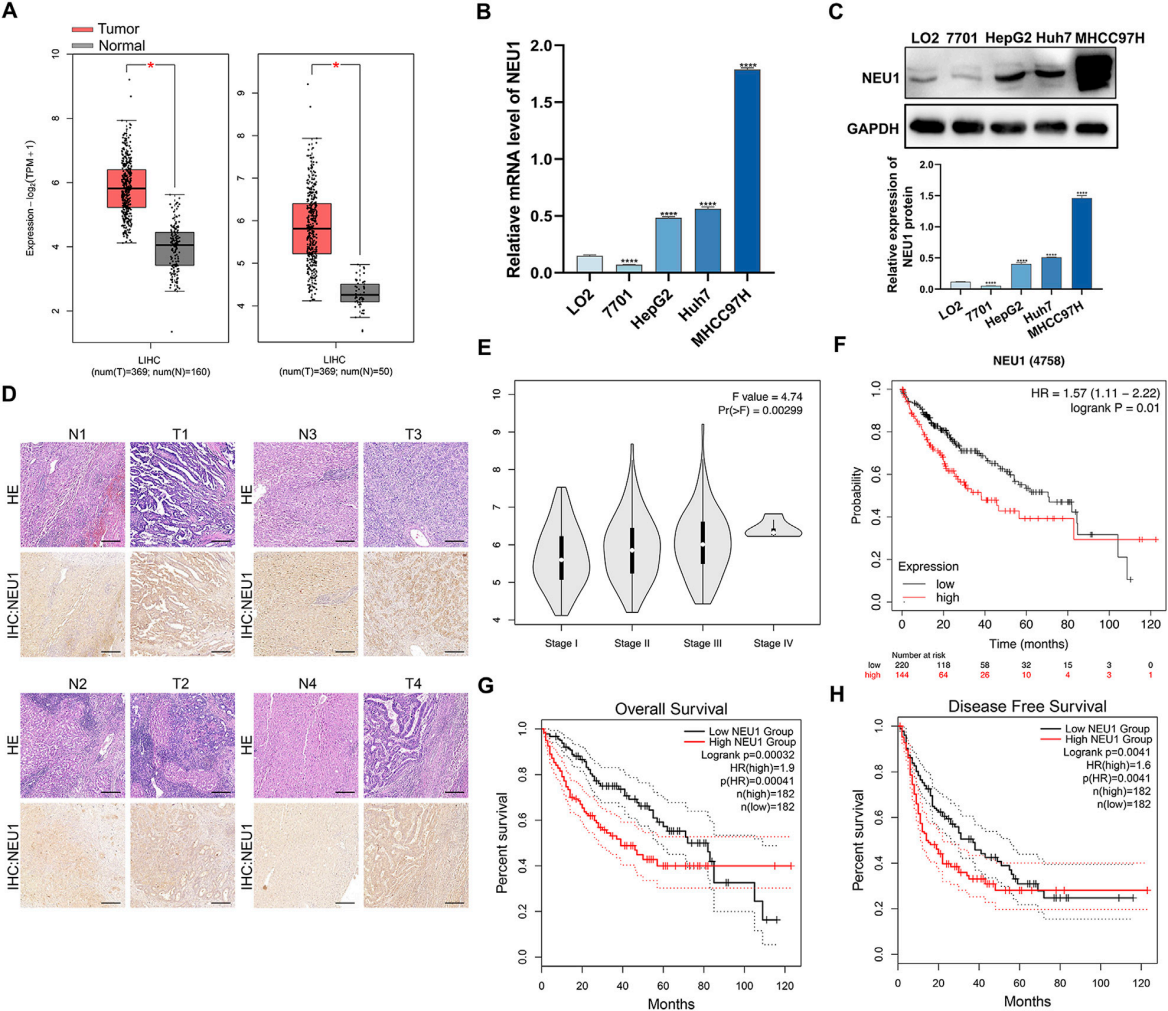
The relationship between the NEU1 expression and clinicopathologic parameters. **(A)** NEU1 expression in the TCGA LIHC cohort. **(B)** qRT-PCR and **(C)** Western blot analysis of NEU1 mRNA and protein expression in liver cancer cell lines and normal human liver cell lines. **(D)** Hematoxylin and eosin staining and IHC analyses were performed with 4 paired tumor tissues and adjacent non-cancerous tissues. Scale bar = 10 μm. **(E)** Relative expression of NEU1 in pathologic stage I, II, III, or IV; **(F–H)** The Kaplan-Meier survival curves comparing liver cancer patients with low (black) and high (red) NEU1 expression were plotted.

We analyzed the expression of NEU1 across various tumor samples using a wide range of databases and analytical tools, including cBioPortal, GEPIA and GEO. Data from the cBioPortal platform revealed that NEU1 was expressed in multiple tumor tissues (Supplementary Figure S1). The GEPIA database showed the NEU1 expression patterns across 33 types of tumor samples and paired normal tissues (Supplementary Figure S2). Furthermore, independent TCGA database analysis and combined Genotype-Tissue Expression data showed that the expression of NEU1 was markedly elevated in liver cancer tissues in comparison to adjacent tissues (Figure 1A). To further validate this finding, we also analyzed six GEO datasets, revealing. remarkable overexpression of NEU1 in liver cancer across the GSE55092, GSE121248, GSE45436, GSE62232, GSE101685, and GSE76427 datasets (Supplementary Figure S3). Then, we examined NEU1 protein levels and mRNA levels in human normal liver cell lines (LO2, QSG-7701) and liver cancer cell lines (HepG2, Huh7, MHCC97H) by Western blot and qRT-PCR. Our findings demonstrated that the NEU1 was overexpressed in liver cancer cell lines, particularly in MHCC97H (Figures 1B, C). Moreover, we measured NEU1 protein expression in 4 paired liver cancer tissues by IHC staining and revealed increased NEU1 expression in tumor tissues compared to matched adjacent tissues (Figure 1D). It is noteworthy that the expression level of NEU1 was associated with disease progression and poor prognosis in liver cancer patients. The elevated expression of NEU1 was found to be significantly correlated to advanced tumor stage (Figure 1E). Survival curves were generated to depict the survival outcome of patients with high or low NEU1 expression. Notably, patients with low NEU1 expression had better prognosis than those with high expression (Figure 1F). Specially, patients with low NEU1 expression exhibited significantly improved overall survival and disease-free survival (Figures 1G, H). These findings indicate that NEU1 is overexpressed in liver cancer, and significantly correlated to the tumor stage and survival status in patients with liver cancer.

### 3.2 NEU1 promotes the proliferation, migration and invasion of liver cancer cell

**FIGURE 2:**
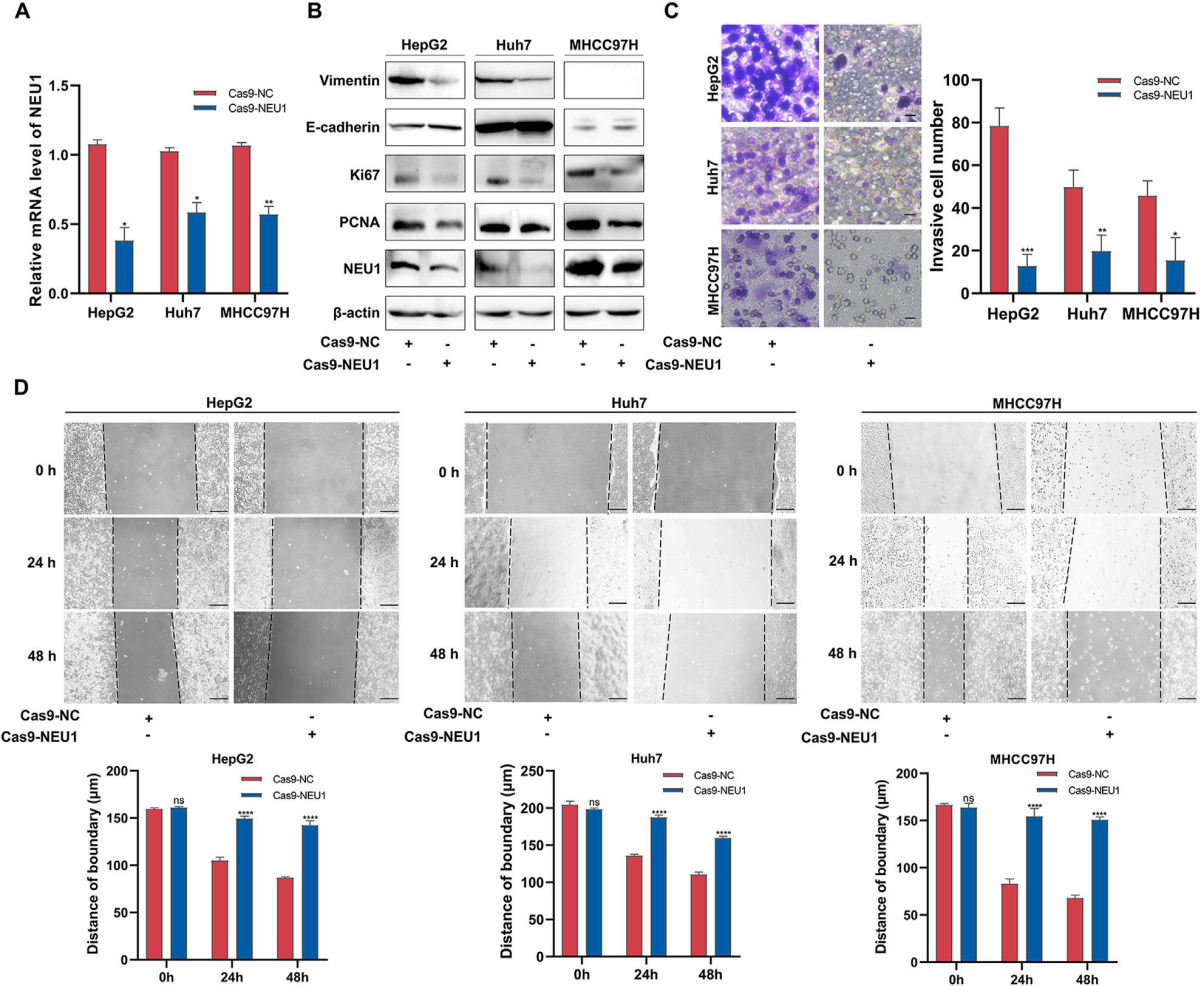
NEU1 promotes the proliferation, migration and invasion of liver cancer cell lines. **(A)** Validation of the knockdown efficiency of NEU1 in liver cancer cell lines transfected with Cas9-NC or Cas9-NEU1 using qRT-PCR. **(B)** The expression of EMT markers in liver cancer cells. **(C)** The invasion ability of liver cancer cell lines detected with transwell assays and **(D)** the migration of liver cancer cells lines examined with scratch assays in Cas9-NC or Cas9-NEU1 group. Transwell scale bar = 10 μm. Scratch assay scale bar = 200 μm *P < 0.05; **P < 0.01; ***P < 0.001; ****P < 0.0001; n. s. not significant.

We used CRISPR/Cas9 to knockdown NEU1, selecting the second sgRNA for follow-up studies due to its higher knockdown efficient (Supplementary Figures S4A, B). We knocked down NEU1 expression in HepG2, Huh7, MHCC97H cells to determine its effect on the malignant phenotype of cancer cell (Figure 2A). Western blot analysis was employed to assess the impact of NEU1 knockdown on proteins related to the epithelial-mesenchymal transition (EMT) signaling pathway (E-cadherin, Vimentin) and tumor cell proliferation (Ki67, PCNA). The results showed a notably decrease in mesenchymal marker protein Vimentin and an elevated level of the epithelial marker protein E-cadherin. Moreover, the levels of Ki67 and PCNA were significantly diminished following NEU1 knockdown (Figure 2B; Supplementary Figure S4C). Furthermore, CCK-8 assay showed that knockdown of NEU1 significantly inhibited the proliferation of the liver cancer cells (Supplementary Figure S4D). Transwell assay results demonstrated that the invasion capability was markedly diminished in all 3 cell lines after NEU1 knockdown (Figure 2C). In addition, results of scratch assay demonstrated a significant inhibition in migration ability of liver cancer cells following NEU1 knockdown (Figure 2D). Collectively, these results suggest that low NEU1 expression is crucial for inhibiting liver cancer proliferation and invasion.

### 3.3 NEU1 regulates lipophagy by interacting with PLIN2, thereby influencing liver cancer progression

**FIGURE 3:**
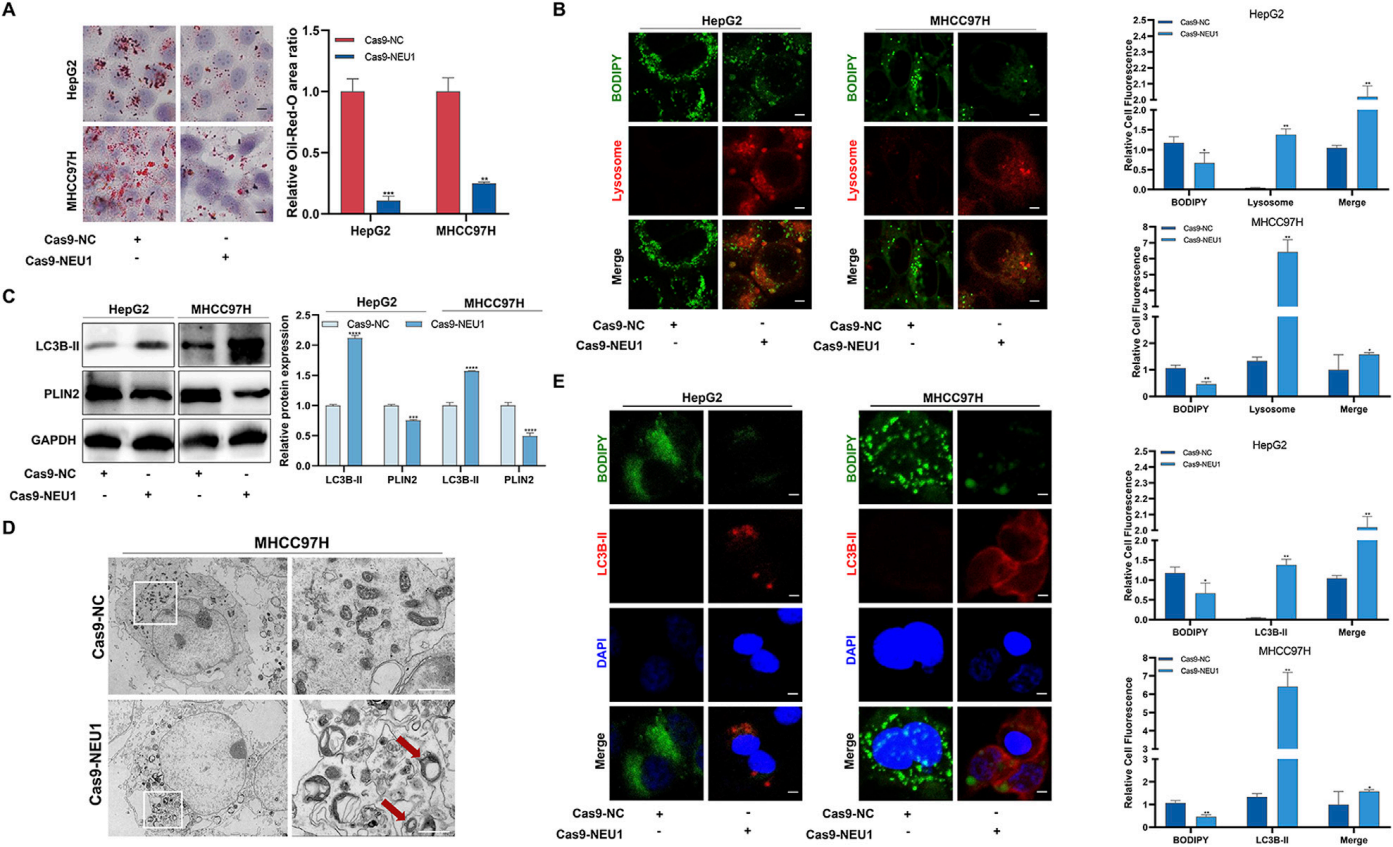
The expression of NEU1 is negatively correlated with the level of lipophagy. **(A)** Effect of NEU1 on lipid droplets in liver cancer cell lines. **(B)** Immunofluorescence analysis of lipid droplets (green) and lysosome markers (red) in HepG2 and MHCC97H cells with NEU1 knockdown. Scale bar = 50 μm. **(C)** The effect of NEU1 on the expression of autophagy marker protein LC3B-II and lipid droplet marker protein PLIN2 detected with Western blot. **(D)** Representative transmission electron micrograph. The red arrow represents a lipid drop swallowed by the autolysosome. Scale bar = 10 μm. **(E)** Immunofluorescence analysis of lipid droplets (green) and autophagy markers (red) in HepG2 and MHCC97H cells with NEU1 knockdown. Scale bar = 20 μm *P < 0.05; **P < 0.01; ***P < 0.001; ****P < 0.0001.

To explore the downstream regulatory mechanisms of NEU1, we employed RNA sequencing to identify differentially expressed genes between the Cas9-NC group and Cas9-NEU1 group. GO enrichment analysis of differentially expressed genes revealed key biological processes, cell components, and molecular functions associated with NEU1 alteration, including lipid-related genes (Supplementary Figure S5A). Given the crucial role of lipophagy in maintaining liver lipid metabolism homeostasis, we performed a correlation analysis using the GEPIA platform, which revealed a positive correlation between NEU1 expression and lipid synthesis-related genes, such as sterol regulatory element-binding protein 1 and fatty acid synthase (Supplementary Figure S5B). In addition, the notable decrease in lipid droplets in the 3 cell lines following NEU1 knockdown suggests involvement of the lysosomal pathway (Figure 3A). To verify that this reduction of lipid droplets is associated with lysosomal-related lipophagy, we performed immunofluorescence analysis on HepG2 and MHCC97H cells before and after NEU1 knockdown. The results showed that NEU1 knockdown inhibited the accumulation of BODIPY-labeled lipid droplets and increased the colocalization of lipid droplets and lysosomes in both cell lines (Figure 3B). The Western blot analysis revealed a decrease in the lipid droplet protein PLIN2 levels and an increase in the autophagy-related protein LC3B-II (Figure 3C), indicating potential induction of lipophagy. Additionally, transmission electron microscopy allowed us to directly observe lipophagy, showing lipid droplets encapsulated within autophagic lysosome in NEU1-knockdown liver cancer cells (Figure 3D; Supplementary Figure S5C). Furthermore, immunofluorescence analysis demonstrated that decreased NEU1 expression resulted in diminished lipid droplets and enhanced autophagy (Figure 3E). All these findings support that reduced NEU1 expression promotes lipohagy and restrains the progression of liver cancer.

**FIGURE 4:**
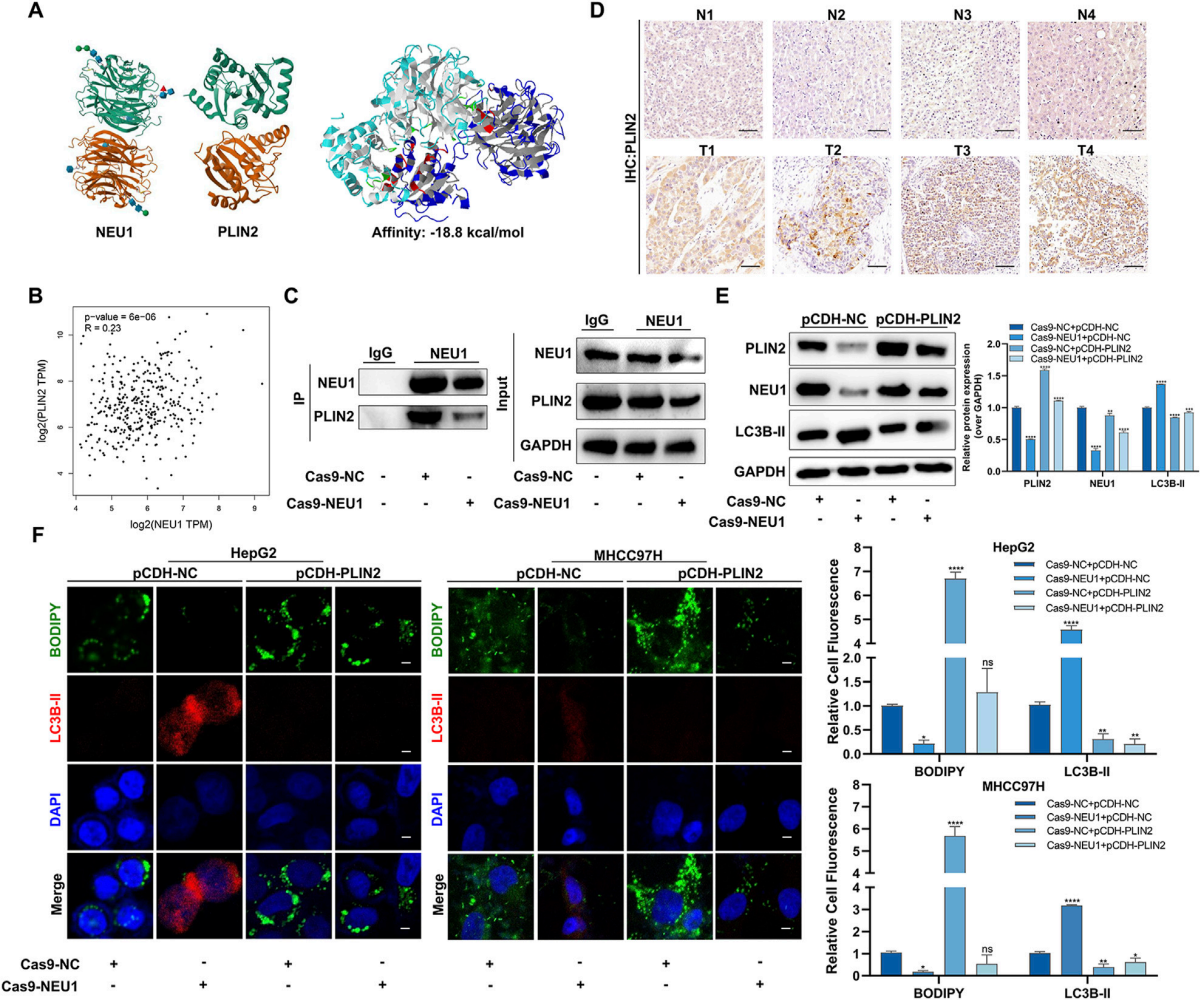
NEU1 binds with PLIN2 to regulate lipophagy. **(A)** The Protein-protein interacting structures of the NEU1 and PLIN2 complex were predicted using ZDOCK. **(B)** The correlation between NEU1 and PLIN2 mRNA in TCGA liver cancer tissues. **(C)** Co-IP experiment was used to verify the binding ability of NEU1 to PLIN2. **(D)** The expression of PLIN2 in 4 paired tumor tissues and adjacent normal tissues was analyzed by IHC analyses. Scale bar = 50 μm. **(E)** Levels of NEU1 and PLIN2 in the MHCC97H were examined by Western blot across the four treatment groups. **(F)** Representative images showing the colocalization of lipid droplets (green) and LC3B-II (red) in groups (Left). Manders’ overlap coefficients for co-localization of lipid droplets or LC3B-II were calculated using IPP 6.0, while the quantification is shown in a bar graph (Right). Scale bar = 20 μm *P < 0.05; **P < 0.01; ***P < 0.001; ****P < 0.0001; n. s. not significant.

Considering that PLIN2 is a lipid metabolism-associated protein that plays an important role in lipid accumulation. We concentrated on the molecular domains to investigate the interaction between NEU1 and PLIN2. The result of ZDOCK showed a potential binding energy of −18.8 kcal/mol between NEU1 and PLIN2, suggesting a strong direct interaction between the two proteins (Figure 4A). Moreover, a positive correlation between the mRNA expression of NEU1 and PLIN2 was observed (Figure 4B). Subsequent co-immunoprecipitation assay verified the direct binding of NEU1 and PLIN2, with a decrease reduced in PLIN2 expression following Cas9-NEU1 treatment (Figure 4C). Immunohistochemistry analysis of the four pairs of clinical liver cancer samples revealed a significantly higher expression of PLIN2 in the cancer tissues compared to adjacent tissues (Figure 4D). Similarly, immunofluorescence analysis confirmed the direct binding of NEU1 and PLIN2, showing a significant reduction in labeled PLIN2 fluorescence intensity upon NEU1 knockdown (Supplementary Figure S6). To assess the role of PLIN2 in NEU1-mediated lipophagy, we overexpressed PLIN2 in HepG2 and MHCC97H cell lines, which led to an inhibition of lipophagy. This inhibition was reversed after Cas9-NEU1 treatment (Figure 4E). Moreover, immunofluorescence analysis revealed that Cas9-NEU1 mitigated lipid autophagy injury induced by PLIN2 overexpression (Figure 4F). These results indicate that PLIN2 is a critical mediator of NEU1-induced lipophagy.

**FIGURE 5:**
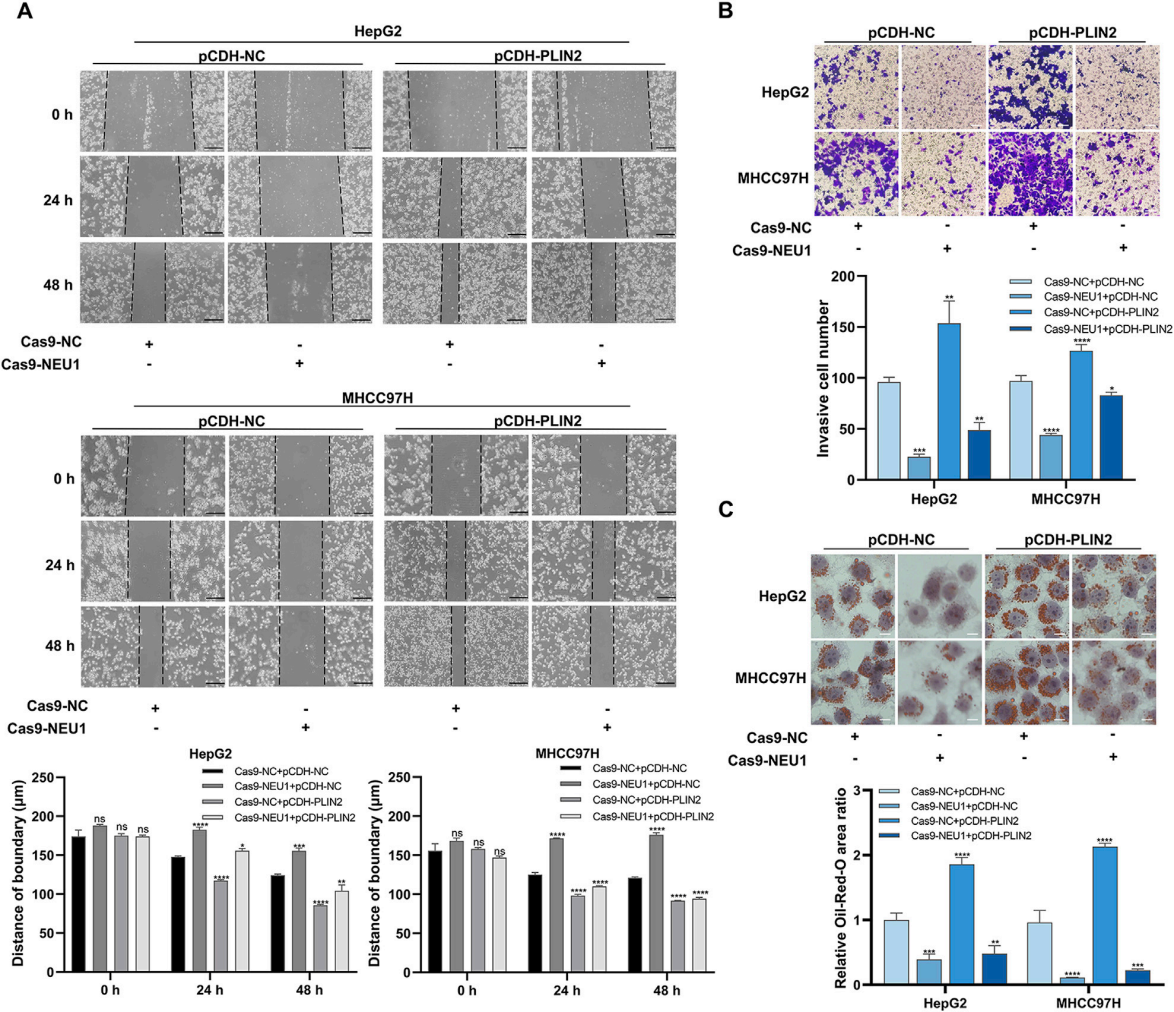
NEU1 regulates liver cancer progression through PLIN2. **(A)** Scratch assay was performed to detect the changes in migration ability. Scale bar = 200 μm. **(B)** Transwell assay was performed to evaluate the changes in invasion ability, and **(C)** Oil red O staining was performed to detect the changes in lipid accumulation. Transwell assay scale bar = 20 μm; Oil red O staining scale bar = 10 μm *P < 0.05; **P < 0.01; ***P < 0.001; ****P < 0.0001; n. s. not significant.

In two liver cancer cell lines (HepG2 and MHCC97H), we verified that NEU1 affected the malignant phenotype of liver cancer via PLIN2. The scratch assay demonstrated that the wound healing rate and cell migration ability were significantly lower in the NEU1 knockdown group compared to the control group. Conversely, PLIN2 overexpression led to an increase in these parameters (Figure 5A). Intriguingly, PLIN2 overexpression reversed the alteration in cell migratory ability after NEU1 knockdown under co-transfection conditions (Figure 5A). Besides, the transwell assay showed that the invasive ability decreased upon NEU1 knockdown, while PLIN2 overexpression significantly increased invasiveness compared to the interference control group (Figure 5B). Oil red O staining indicated that PLIN2 overexpression rescued the reduced intracellular lipid accumulation induced by NEU1 knockdown (Figure 5C). In summary, NEU1 knockdown inhibits liver cancer progression by reducing PLIN2 expression.

### 3.4 Oseltamivir phosphate inhibits the proliferation, invasion and migration of liver cancer

**FIGURE 6:**
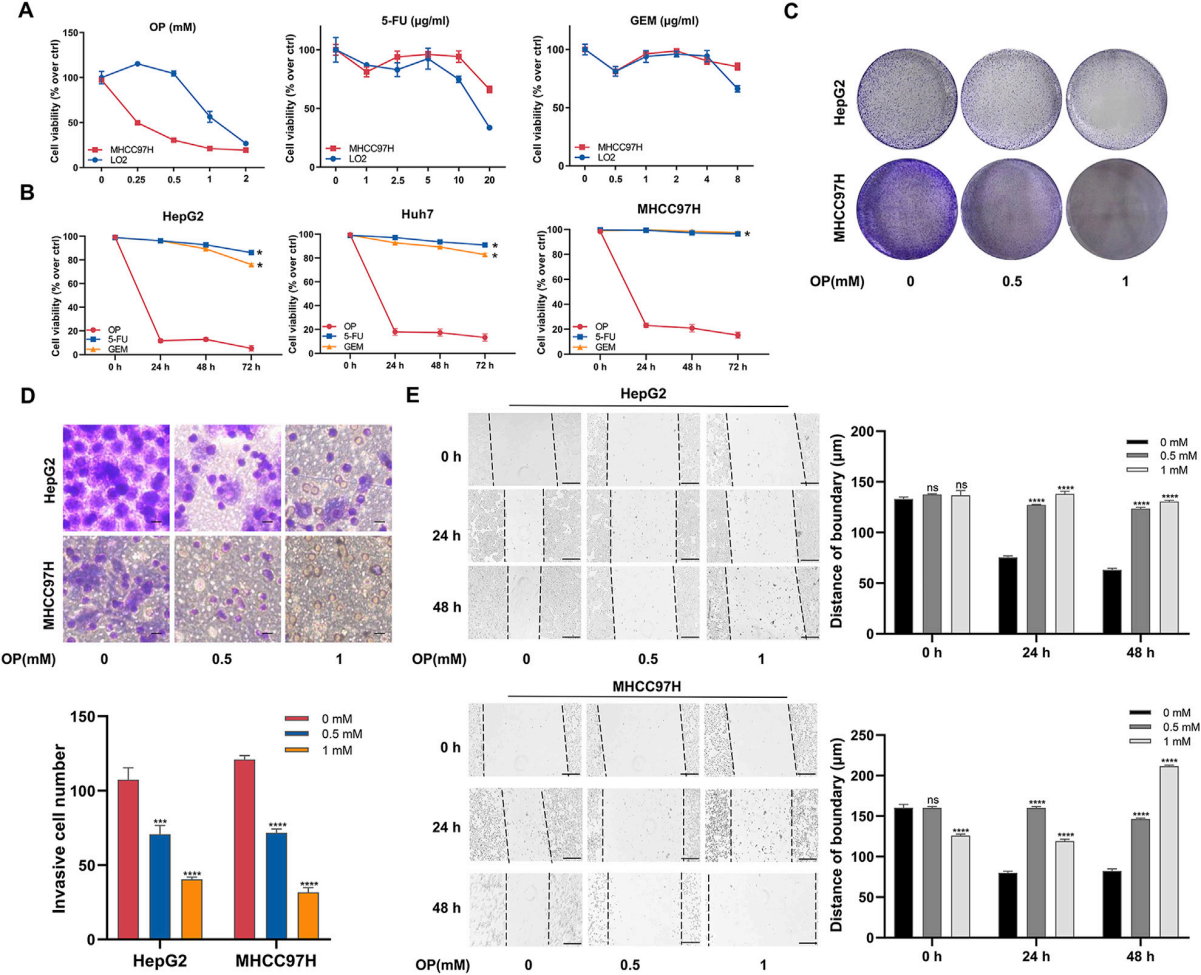
Oseltamivir phosphate inhibits the proliferation, invasion and migration of liver cancer cells. **(A)** The impact of varying concentrations of OP, 5-FU, and GEM on the viability of liver cancer cells and human normal liver cell. **(B)** The effect of OP, 5-FU and GEM on the viability of liver cancer cells. **(C)** The effect of different concentrations of OP on the proliferation of liver cancer cells as examined with plate clone formation assays. **(D)** The effect of different concentrations by OP on the invasion of liver cancer cells as detected with transwell assays. Scale bar = 10 μm. **(E)** The effect of different concentrations of OP on the migration of liver cancer cells as examined with scratch assays. Scale bar = 200 μm *P < 0.05; ***P < 0.001; ****P < 0.0001; n. s. not significant.

OP functions act as a specific inhibitor of neuraminidase, targeting NEU1, and has shown promising potential in cancer treatment. We further explored whether OP has cytotoxic effects on liver cancer. By varying the concentrations of the OP and other two clinical drugs with high drug resistance (5-FU and GEM), MHCC97H cells showed greater sensitivity to OP treatment. Additionally, OP exhibited less toxicity to normal liver cell (LO2) at a concentration of 0.5 mM. This concentration was selected for subsequent experiments (Figure 6A). In the drug resistance test, HepG2, Huh7 and MHCC97H cells demonstrated greater sensitivity to OP compared to 5-FU and GEM (Figure 6B). Besides, the results of clone formation assay, transwell assay, and scratch assay indicated that OP reduced cell proliferation, invasion, and migration in a dose-dependent manner in both liver cancer cell lines (Figures 6C–E).

### 3.5 Oseltamivir phosphate induces lipophagy in liver cancer through NEU1-PLIN2 axis *in vitro* and *in vivo*

**FIGURE 7:**
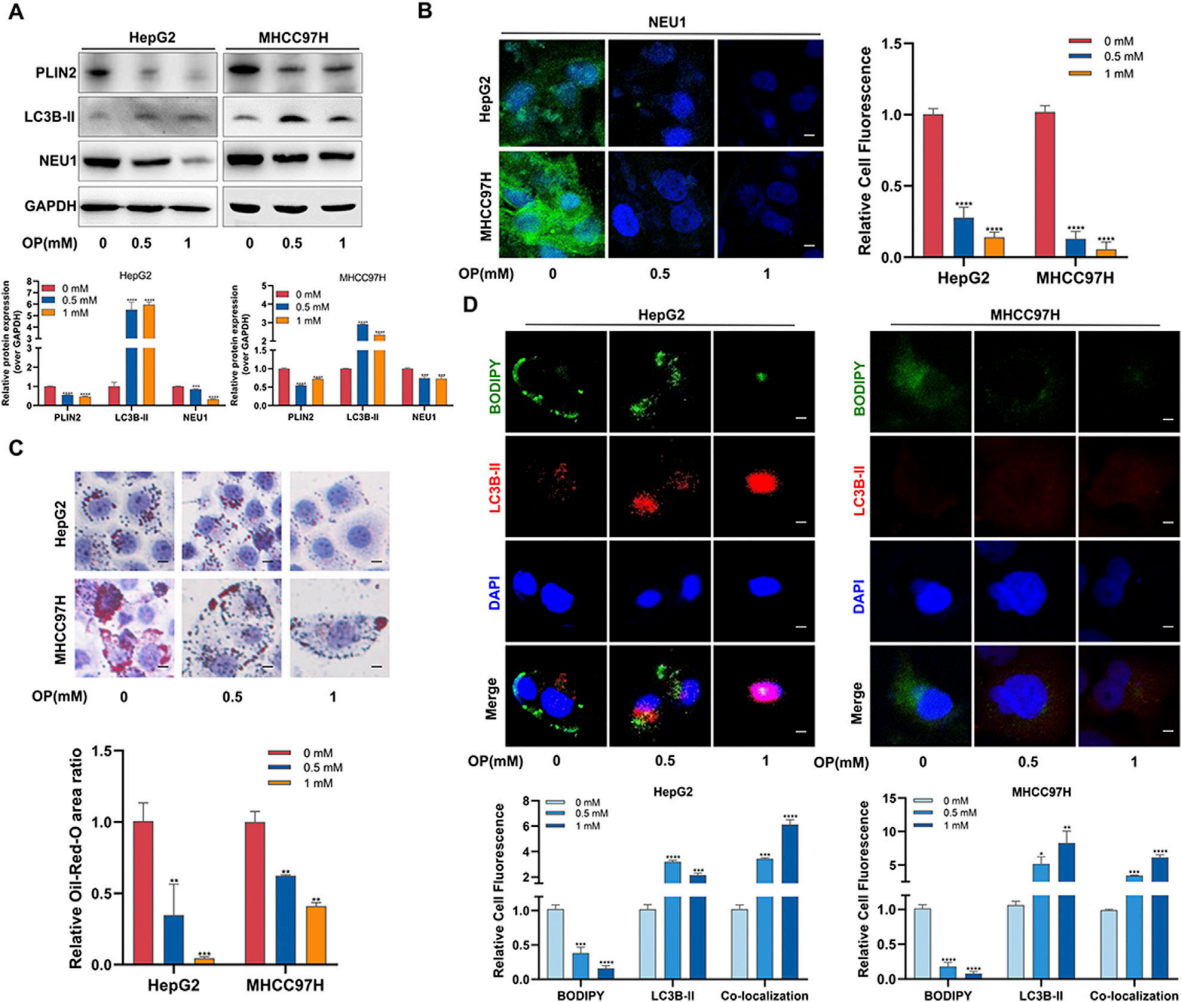
Oseltamivir phosphate induces lipophagy in liver cancer cells through NEU1-PLIN2 axis. **(A)** Western blot was used to detect the changes of lipophagy-related proteins in HepG2 and MHCC97H cells treated with different concentrations of OP. **(B)** Immunofluorescence assay was used to detect the changes of NEU1 in HepG2 and MHCC97H cells treated with different concentrations of OP. Scale bar = 50 μm. **(C)** Lipid accumulation levels in HepG2 and MHCC97H cells treated with different concentrations of OP were detected by oil red O staining. Scale bar = 10 μm. **(D)** The changes of lipophagy in HepG2 and MHCC97H cells treated with different concentrations of OP were detected by immunofluorescence. Scale bar = 20 μm *P < 0.05; **P < 0.01; ***P < 0.001; ****P < 0.0001.

Buliding upon previous findings that NEU1 knockdown promotes lipophagy and inhibits liver cancer by reducing PLIN2, we then investigated the effect of OP on lipophagy in liver cancer. Consistent with the Cas9-NEU1 results, OP inhibited the accumulation of BODIPY-labeled lipid droplets and increased the colocalization of lipid droplets with lysosomes in HepG2 and MHCC97H cells (Supplementary Figure S7A). Electron microscopy confirmed increased lipophagy in liver cancer cells following OP treatment (Supplementary Figure S7B). Western blot and immunofluorescence analyses showed that OP treatment significantly reduced NEU1 expression (Figures 7A, B). In addition, the number of lipid droplets was markedly reduced in a dose-dependent manner after OP treatment (Figure 7C). Western blot and immunofluorescence analyses revealed that OP inhibited PLIN2 protein expression and fluorescence intensity while significantly increasing the autophagy marker LC3B-II (Figures 7A, D). Altogether OP could effectively treat liver cancer by inducing NEU1-mediated lipophagy.

**FIGURE 8:**
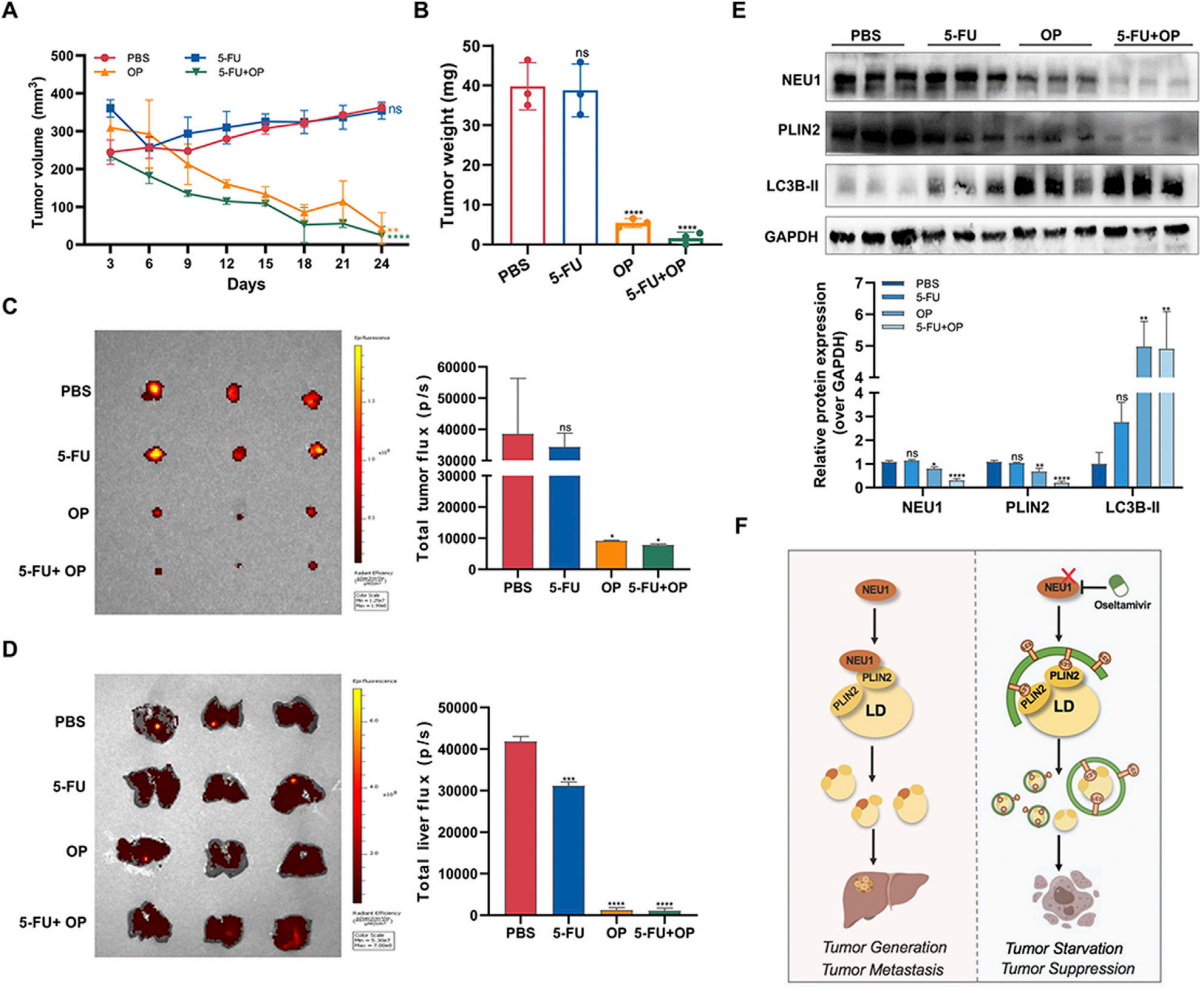
Oseltamivir phosphate inhibits the growth and metastasis of subcutaneous tumors in nude mice. **(A, B)** The effects of OP and 5-FU alone or in combination on the volume and weight of subcutaneous tumors in nude mice. **(C)** Subcutaneous tumor growth in nude mice was monitored by *in vivo* imaging of small animals. **(D)** The livers of tumor-bearing mice were monitored using *in vivo* imaging of small animals. **(E)** Detection of the changes of NEU1 and lipophagy-related proteins levels in subcutaneous tumor tissues of nude mice after drug treatment by Western blot. **(F)** OP, as a NEU1 inhibitor, promote lipophagy through regulating the NEU1-PLIN2 signaling axis, thereby exerting anti-liver cancer effects. *P < 0.05; **P < 0.01; ***P < 0.001; ****P < 0.0001. n. s. not significant.

To further assess the inhibitory effect of OP on liver cancer, we established mouse xenograft tumor models by subcutaneously injecting MHCC97H cells into athymic nude mice. The results showed that OP significantly impeded tumor growth, with a more pronounced effect observed when used in combination with 5-FU (Figures 8A–C). Metastasis of malignant tumors often indicates treatment failure and represents a principal cause of mortality among cancer patients. Our investigation showed that the incidence of liver metastases was notably reduced with OP, either alone or in combination therapy, compared to treatment with 5-FU alone (Figure 8D). This supports the effectiveness of OP and combination therapy in mitigating xenograft tumors growth and metastasis. Consistent with cellular level findings, OP inhibited NEU1 expression and promoted lipophagy in tumor-bearing mice (Figure 8E). In summary, NEU1-induced elevation of PLIN2 leads to lipid accumulation, which promote tumorigenesis and metastasis of liver cancer. OP targets NEU1, promoting degradation of lipid droplet and enhancing autophagy, thereby counteracting these processes (Figure 8F).

## 4 Discussion

In the present study, we evaluated NEU1 expression characteristics in liver cancer based on public databases and independent cohorts, demonstrating a significant upregulation of NEU1 in liver cancer cells and tissues. Besides, higher NEU1 expression was linked to disease progression and survival outcomes in liver cancer patients. In addition, our findings revealed that NEU1 knockdown reduced proliferation, invasion, and migration of liver cancer cells. Furthermore, functional studies revealed that NEU1 regulated lipophagy by interacting with PLIN2, thereby involving in the progression of liver cancer. Cell and animal experiments showed that OP, as a NEU1 inhibitor, promote lipophagy through regulating the NEU1-PLIN2 signaling axis, thereby exerting anti-liver cancer effects.

Accumulating evidence demonstrates that NEU1 regulates sialylation of key proteins to control several biological processes including cell proliferation, differentiation, and apoptosis (Haxho et al., 2016; Glanz et al., 2019). Elevated NEU1 expression has been associated with several types of cancers. For example, the expression of NEU1 in melanoma tissues was found to be markedly elevated in comparison to that observed in adjacent tissues (Peng et al., 2022). Inhibition of NEU1 expression can suppress ovarian cancer proliferation, apoptosis and invasion (Ren et al., 2016). In non-small cell lung cancer, NEU1 expression is elevated and correlates to a poor clinical prognosis (Lv et al., 2020). Conversely, NEU1 has been reported to suppress metastasis in colon cancer both *in vitro* and *in vivo* (Bansal et al., 2014). In bladder cancer, the inhibition of cancer cell proliferation, induction of apoptosis and suppression of tumor formation have all been shown to be facilitated by the action of NEU1 (Zhou et al., 2020). Currently, the function of NEU1 in various tumors remains controversial.

Our results indicated that NEU1 is remarkably upregulated in liver cancer patients and showed negative relationship with clinical stage and survival prognosis. These findings suggest NEU1 may be used as a prognostic marker for liver cancer. Although, significant links were observed between NEU1 expression and liver cancer progression in our study and previous bioinformatic study (Wu et al., 2021). However, the biological function and associated mechanisms of NEU1 in liver cancer remain poorly elucidated. The diversity of NEU1 substrates allows it to influence various signal transduction pathways. NEU1 is recognized as a modulator of cell receptors and is involved in endocytosis and autophagy through lysosomal pathways (Pshezhetsky and Hinek, 2011; Allendorf and Brown, 2022; Chen et al., 2020). For example, desialylation of sialyl a-2,3-linked β-galactosyl residues of Toll-like receptor 4 by NEU1 was essential for receptor activation and cellular signaling via NF-kB activation in dendritic and macrophage cells (Debnath, 2011; Seyrantepe et al., 2010). Decreased expression or function of NEU1 reduced atherosclerosis in mice through its significant effects of non-high-density lipoprotein cholesterol on lipid metabolism and inflammatory processes (White et al., 2018). In addition, it has been shown that elevated NEU1 activity has been observed in hepatic epididymal fat of obese mice (Natori et al., 2013), and sialidase inhibitors have been shown to mitigate high fat diet-induced adipose tissue mass, liver inflammation and steatosis in mice (Pilling et al., 2021).

In our study, RNA-sequencing results revealed a close association between NEU1 and lipid metabolism in liver cancer. Moreover, knockdown of NEU1 led to increased colocalization of lipid droplets with lysosomes and enhanced levels of the autophagy marker protein LC3B-II. This observation suggests that NEU1 may be involved in endocytosis and autophagy via lysosomal pathway. Sing et al*.* found that starvation-induced autophagy contributes to lipid droplet degradation in RALA255-10G rat hepatocytes and proposed the term “lipophagy” (Singh et al., 2009). Lipophagy occurs within lysosome and involves autophagy isolation of lipid droplets in the autophagosome, which then fuse with lysosome. The lipid droplets are ultimately degraded by lysosomal lipase, with both lipolysis and lipophagy being activated simultaneously. However, the molecular mechanism by which NEU1, a lysosomal enzyme, regulates lipophagy remains unclear.

Recent studies have highlighted the importance of PLIN2 degradation in reducing hepatic lipid accumulation through activated lipophagy. Irungbam et al*.* found that the reduced PLIN2 expression promotes the colocalization of LC3B-II and lysosomal-associated membrane protein 1on the lipid droplet surface, thereby triggering lipophagy and mitigating hepatic steatosis (Irungbam et al., 2020). Similarly, Martinez-Lope et al. found that cold exposure to 4°C, compared with room temperature, enhanced LC3B-II localization on the lipid droplet in the liver of C57BL/6 mice, which alleviated lipid accumulation by activating lipophagy (Martinez-Lopez et al., 2016). As previously mentioned, we found a significant reduction in lipid droplets and an increase in autophagy in the NEU1 knockdown liver cancer cells, suggesting the occurrence of lipophagy. To investigate the underlying molecular mechanism, we examined how NEU1-mediated lipophagy influences lipid droplet degradation. Our results revealed that NEU1 directly binds to PLIN2. Decreased NEU1 levels were associated with reduced PLIN2 protein expression and elevated levels of autophagy and lipophagy. In addition, NEU1 knockdown significantly inhibited the liver cancer cells proliferation, invasion and migration by promoting lipophagy through the suppression of PLIN2.

Chemotherapy drugs resistance remians a major factor contributing to adverse clinical outcome in liver cancer patients (Bao and Wong, 2021; Pranzini et al., 2022). 5-FU and GEM are first-line chemotherapy drugs often used for liver cancer. However, the emergence of 5-FU resistance greatly limits its anticancer effectiveness (Zhang et al., 2018; Liu et al., 2022). Similarly, despite GEM is widely used, research shows that up to 70% of cholangiocarcinoma patients exhibit poor responses to GEM treatment (Macias, 2014; Okamoto et al., 2013; Lu et al., 2018). In recent decades, alternative approaches using non-oncological drugs to treat cancer have attracted considerable attention (Armando et al., 2020; Nunes et al., 2020). OP is an antiviral drug, has recently been investigated as a potential alternative therapeutic agent for drug-resistant tumors (Sambi et al., 2020; O'Shea et al., 2014; Itakura and Mizushima, 2011; Yang et al., 2024; Qorri et al., 2022). The current findings have shown that OP may inhibit tumor progression, chemotherapy resistance and metastasis by interfering with the EMT program and cancer stem cell enrichment, and then enhance the efficacy of chemotherapy drugs (Haxho et al., 2014; Qorri et al., 2022; Chuang et al., 2024). A recent study reported that OP can induce apoptosis in Huh-7 cells and both apoptosis and autophagy in HepG2 cells (Huang et al., 2021). However, the exact mechanism of by which OP targets NEU1 to inhibit liver cancer remains unclear. This study revealed that OP exerts a pronounced inhibitory effect on the liver cancer proliferation, invasion and metastasis *in vivo* and *in vitro*. In addition, our findings suggest that OP enhances the sensitivity of liver cancer cells to 5-FU and GEM. The synergistic effect of OP with 5-FU on liver cancer was particularly pronounced. We elucidated that OP targets NEU1, inhibiting PLIN2 and thereby promoting lipophagy, which exerts an antitumor effect. Our study provides new evidence supporting the use of OP to target NEU1 in the treatment of liver cancer, highlighting the critical role of NEU1-PLIN2 mediated lipophagy in liver cancer. It is noteworthy that a previous study demonstrated that OP inhibits the release of inflammatory factors and inflammatory responses by inhibiting NEU1 activity and reducing the hydrolysis of sialic acid on the ends of cell surface glycoproteins (Haxho et al., 2016). Research on the use of anti-inflammatory drugs in liver cancer treatment has garnered increasing attention (Tong et al., 2025; Yeo et al., 2024; Nigam et al., 2023). These findings suggest that the anti-inflammatory properties of OP could offer a promising approach to enhance the therapeutic efficacy in liver cancer treatment.

It is important to note that the present study has some limitations. First, while we provide preliminary evidence that NEU1 interacts with PLIN2, the exact mechanisms through which NEU1 knockdown leads to PLIN2 downregulation require further investigation. Second, additional research is necessary to clarify the molecular mechanism underlying the inhibition of liver cancer through the upregulation of lipophagy. Third, although the significant effects of the NEU1 inhibitor against liver cancer has been observed in both *in vivo* and *vitro* studies, extensive clinical evidence is needed to confirm the efficacy of OP as a treatment for drug-resistant liver cancer. Moreover, recent advancements in nanotechnology have greatly improved drug delivery for liver diseases, enhancing drug stability, bioavailability, and cellular uptake efficiency (Singh et al., 2023; Liu et al., 2024). Future studies should explore the potential of nanocoated OP in liver cancer treatment, which could offer more targeted and effective therapeutic solutions for liver diseases.

In conclusion, our findings suggest that NEU1 plays an oncogenic role in liver cancer by interacting with PLIN2 to regulate lipophagy. Inhibition of NEU1 using OP presents a promising strategy to enhance the efficacy of treatments for drug-resistant liver cancer. Our study not only identifies NEU1 as a potential biomarker and therapeutic target for liver cancer but also provides evidence supporting the use of OP as a treatment option for cancer. Future studies are needed to further validate the anti-liver cancer efficacy of OP and explore its potential in combination with other anti-tumor agents.

## Data Availability

The original RNA-sequencing data presented in this study have been deposited in the NCBI repository under accession number PRJNA1232068. The GSE datasets used in this study are available in the GEO online repositories, and details can be found in Section 2.1 of the article.

## Abbreviations

NEU1, neuraminidase-1; OP, oseltamivir phosphate; IHC, immunohistochemistry; Co-IP, co-immunoprecipitation; PLIN2, perilipin 2; GEO, Gene Expression Omnibus; TCGA, The Cancer Genome Atlas; 5-FU, 5-fluorouracil; IPP 6.0, Image-Pro Plus 6.0; GEPIA, Gene Expression Profiling Interactive Analysis; FBS, fetal bovine serum; qRT-PCR, quantitative real-time PCR; EMT, epithelial-mesenchymal transition; GEM, gemcitabine.
