# Supplementary material for: New use of an old drug: mechanism of oseltamivir phosphate inhibiting liver cancer through regulation of lipophagy via NEU1
Source: Front Pharmacol. 2025 Mar 24;16:1556661. doi: 10.3389/fphar.2025.1556661 (PMC11973263; doi:10.3389/fphar.2025.1556661)
Supplement: Supplementary file 1 [file Image1.pdf]

Supplementary Material

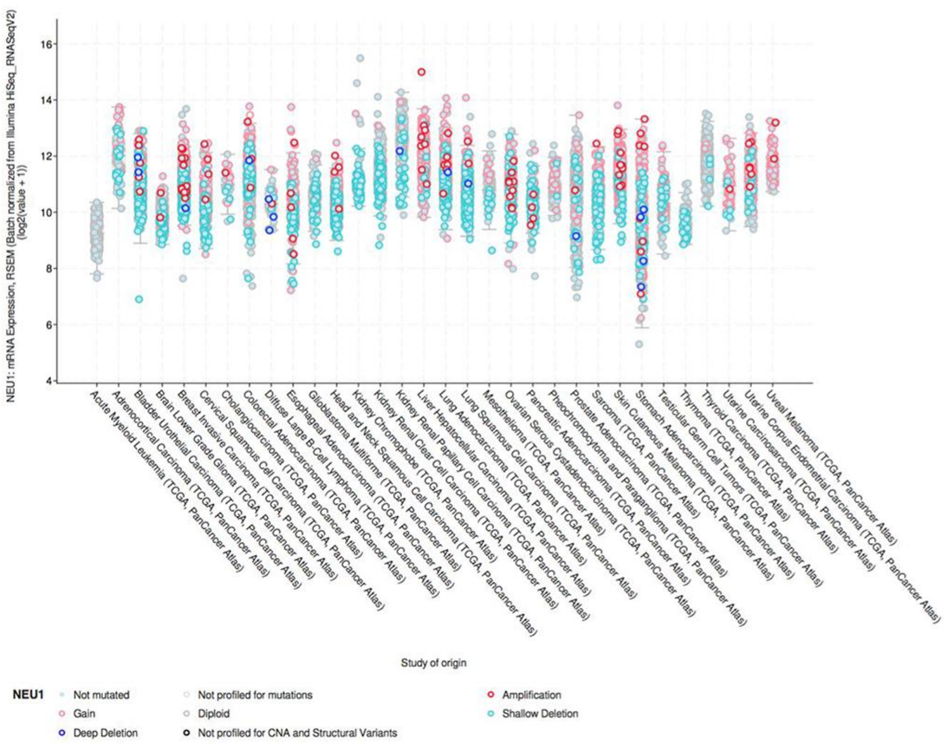

Supplementary Figure 1. The mRNA expression levels of NEU1 in different tumor tissues were analyzed by cBioPortal platform based on TCGA database.

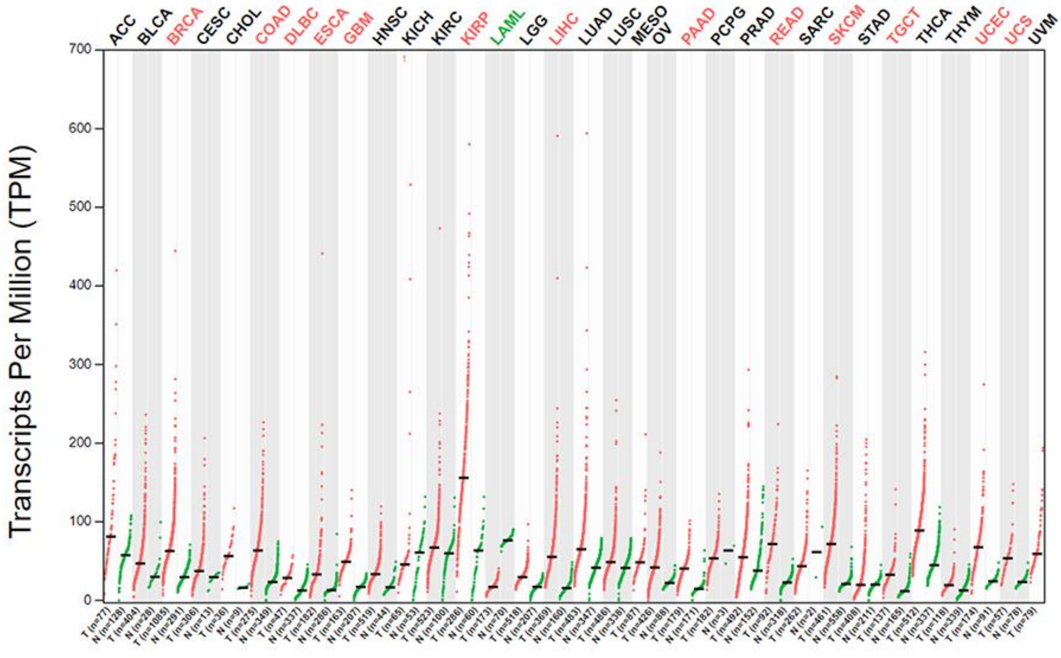

Supplementary Figure 2. Differential expression of NEU1 in tumor and adjacent tissues across 33 different tumors types.

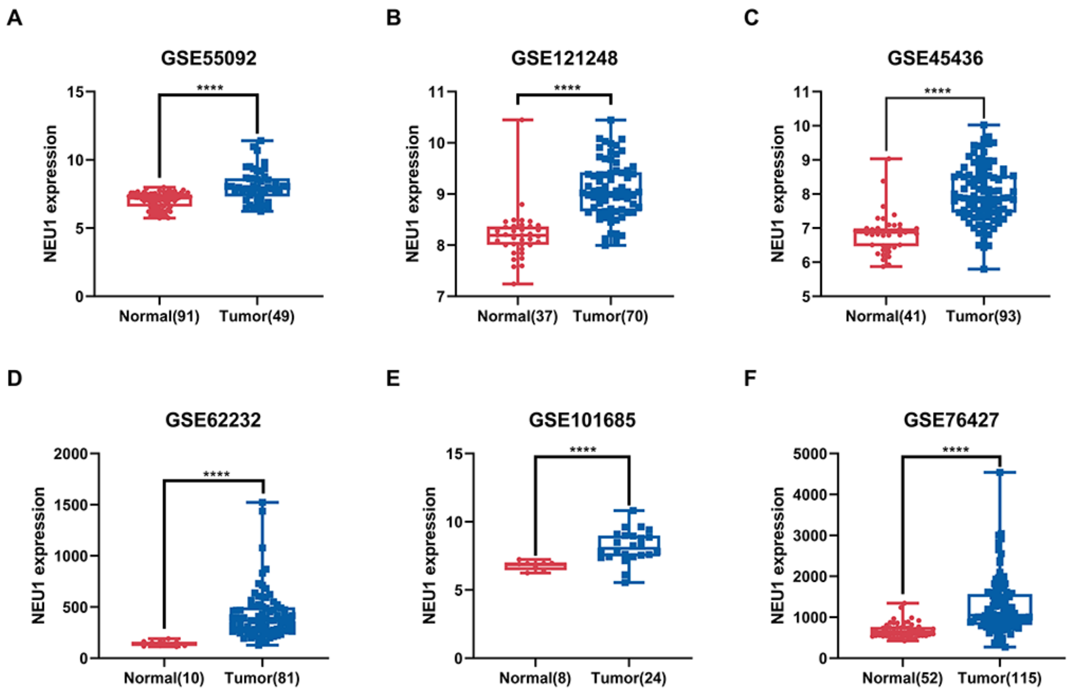

Supplementary Figure 3. Expression of NEU1 in liver cancer and normal tissues in GEO datasets. **(A)** GSE55092 dataset; **(B)** GSE121248 dataset; **(C)** GSE45436 dataset; **(D)** GSE62232 dataset; **(E)** GSE101685 dataset; **(F)** GSE76427 dataset. ****, $P < 0.0001$.

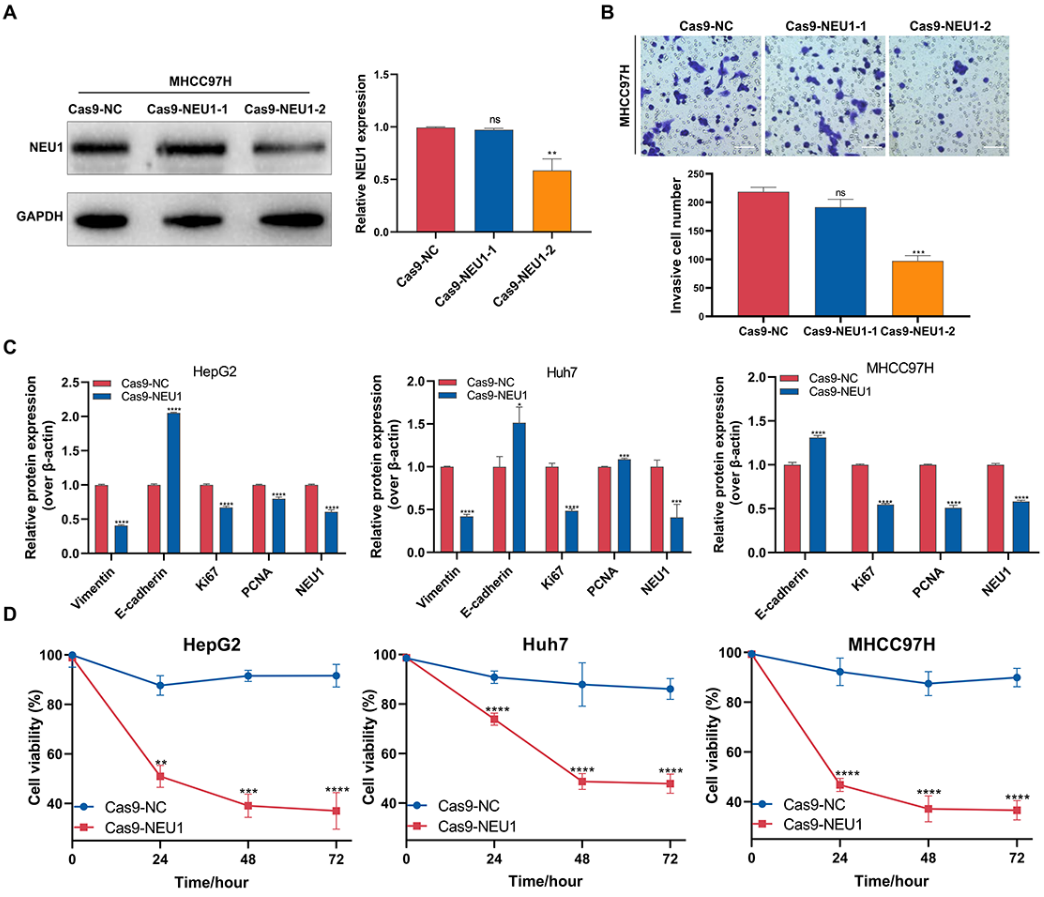

Supplementary Figure 4. Constructing CRISPR-Cas9 cell model and the effect of NEU1 knockdown on proliferation, invasion of the liver cancer cells. **(A)** Validation of the knockdown efficiency of NEU1 in MHCC97H cells transfected with Cas9-NEU1-1 or Cas9-NEU1-2, Cas9-NC using Western blot. **(B)** Effects of NEU1 knockdown on MHCC97H cell invasion were evaluated by transwell assays. **(C)** Quantitative analysis of target proteins in Fig. 2B. **(D)** Effect of NEU1 knockdown on liver cancer cells viability. Scale bar = 20 μm. *, $P < 0.05$; **, $P < 0.01$; ***, $P < 0.001$; ****, $P < 0.0001$; n.s. not significant.

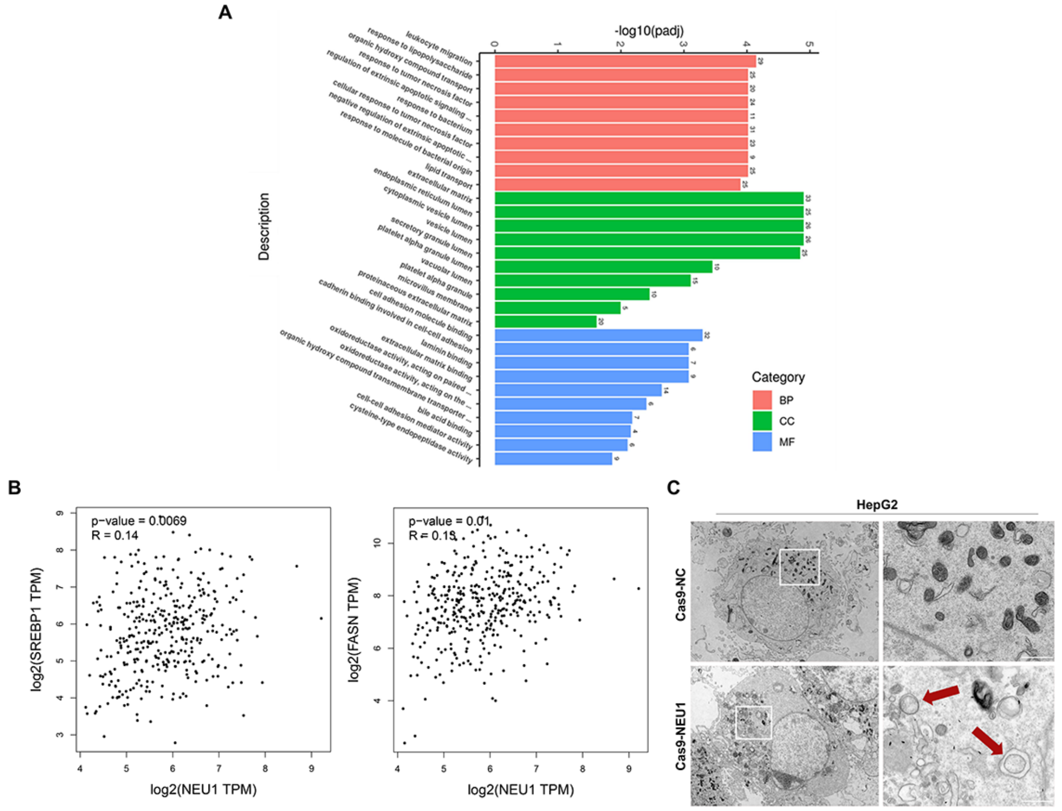

Supplementary Figure 5. The relationships between NEU1 and lipophagy. **(A)** GO enrichment analysis for biological processes, cell components, and molecular functions, respectively. **(B)** Correlation analysis of NEU1 and lipid-related genes. **(C)** Representative transmission electron micrograph of HepG2 cell line. The red arrow represents a lipid drop swallowed by the autolysosome. Scale bar = 10 μm.

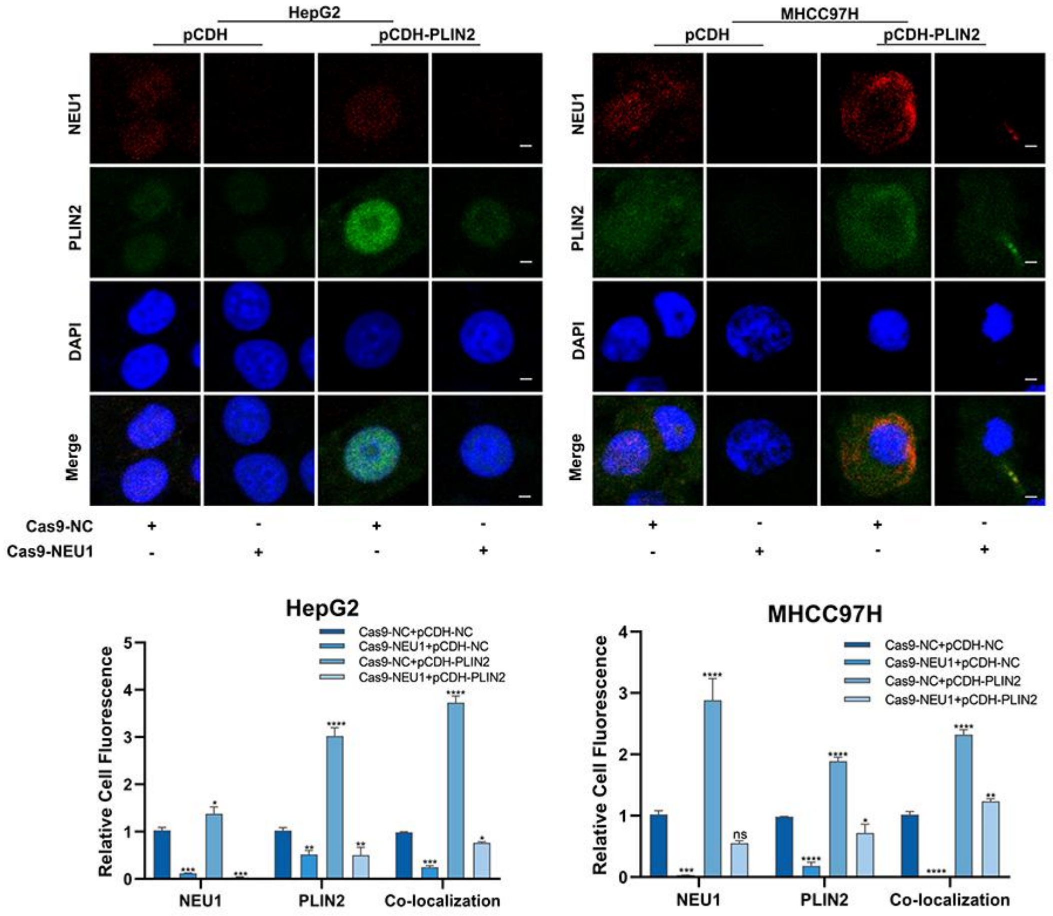

Supplementary Figure 6. Representative images showing the colocalization of NEU1 (red) and PLIN2 (green) were captured by confocal microscopy (top panel). Manders' overlap coefficients for co-localization of NEU1 or PLIN2 were calculated using IPP 6.0, while the quantification is shown in a bar graph (bottom panel). Scale bar = 20 μm. *, $P < 0.05$; **, $P < 0.01$; ***, $P < 0.001$; ****, $P < 0.0001$; n.s. not significant.

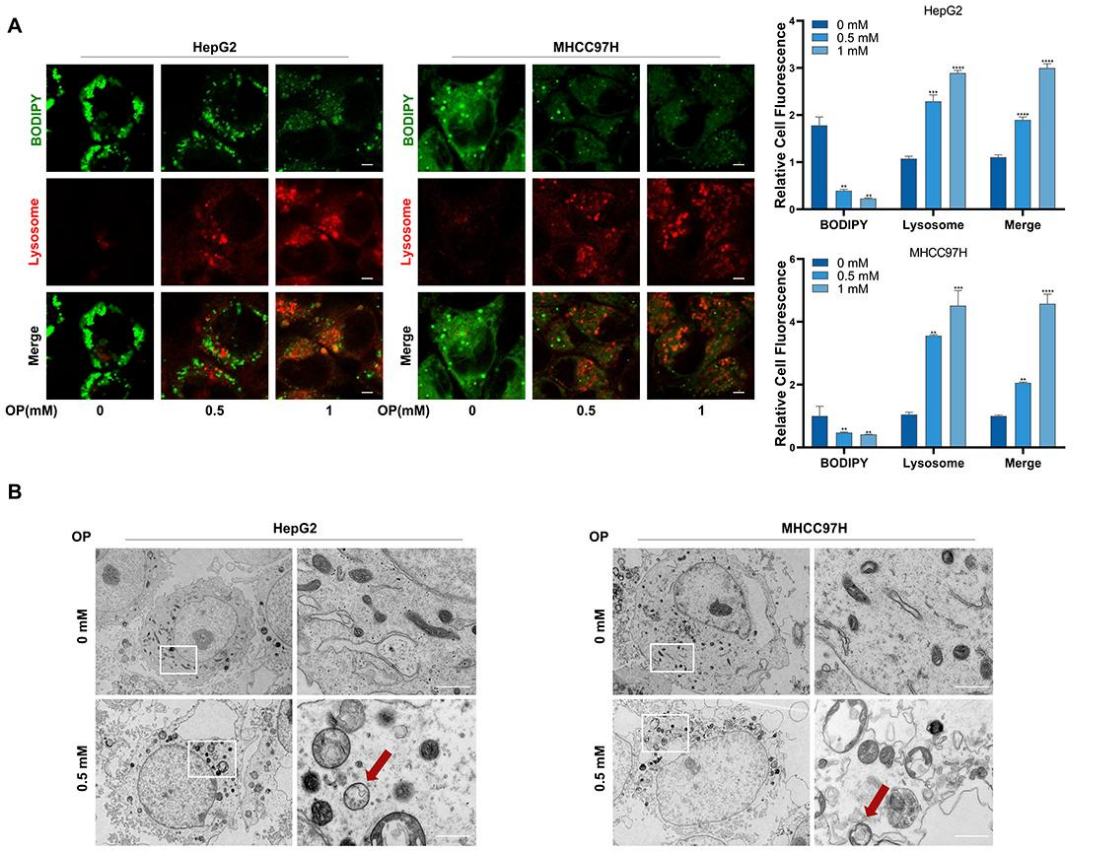

Supplementary Figure 7. The effect of OP treatment on the lipophagy. **(A)** Immunofluorescence analysis of lipid droplets and lysosome markers in HepG2 and MHCC97H cells with OP treatment. Scale bar = 20 μm. **(B)** Representative transmission electron micrograph with OP treatment. Scale bar = 10 μm. **, $P < 0.01$; ***, $P < 0.001$; ****, $P < 0.0001$.
